# Commission Agency for Professional Business

**Published:** 1846-07

**Authors:** 


### COMMISSION AGENCY FOR PROFESSIONAL BUSINESS.

Some of our readers may be served by the publication of the following notice:

"*Wm. A. Clendinen, A.M., M.D.*, of Baltimore, Md., intending to proceed at once to Europe, for professional purposes, will establish, in Paris, a Commission Agency, for the purchase of Anatomical Preparations, Models, Casts; Surgical Instruments and Appliances; Scientific Apparatus; Medical, Surgical, and Scientific Books, Plates, and Engravings; matters of professional and artistic interest. The importance of the Schools of Pathology in Paris, and the advantages arising from them for obtaining the most perfect specimens of Morbid Anatomy, are too generally known to need comment.

"Letters should be most legibly directed to Dr. Wm. A. Clendinen, care of Messrs. A. Bininger & Co., New York, or to Messrs. Clark & Kellogg, Baltimore, who will promptly forward them. In no case will orders be attended to, when unaccompanied by satisfactory remittances, for the amount of which, either of the above firms will receipt; but all cash orders, (howsoever small,) will, when explicitly detailed in writing, be promptly executed. The average commission will not exceed 19 per cent.

"Dr. Clendinen offers his professional services to his countrymen abroad."

April 15th, 1846.

